# Cancer and central nervous system disorders: protocol for an umbrella review of systematic reviews and updated meta-analyses of observational studies

**DOI:** 10.1186/s13643-017-0466-y

**Published:** 2017-04-04

**Authors:** Ferrán Catalá-López, Brian Hutton, Jane A. Driver, Matthew J. Page, Manuel Ridao, José M. Valderas, Adolfo Alonso-Arroyo, Jaume Forés-Martos, Salvador Martínez, Ricard Gènova-Maleras, Diego Macías-Saint-Gerons, Benedicto Crespo-Facorro, Eduard Vieta, Alfonso Valencia, Rafael Tabarés-Seisdedos

**Affiliations:** 1 Department of Medicine, University of Valencia/INCLIVA Health Research Institute and CIBERSAM, Valencia, Spain; 2 Fundación Instituto de Investigación en Servicios de Salud, Valencia, Spain; 3 Clinical Epidemiology Program, Ottawa Hospital Research Institute, Ottawa, Ontario Canada; 4 School of Epidemiology, Public Health and Preventive Medicine, University of Ottawa, Ottawa, Ontario Canada; 5 Geriatric Research Education and Clinical Center, VA Boston Healthcare System, Boston, MA USA; 6 Division of Aging, Department of Medicine, Brigham and Women’s Hospital, Harvard Medical School, Boston, MA USA; 7 School of Public Health and Preventive Medicine, Monash University, Melbourne, Victoria Australia; 8 School of Social and Community Medicine, University of Bristol, Bristol, UK; 9 Instituto Aragonés de Ciencias de la Salud, Red de Investigación en Servicios de Salud en Enfermedades Crónicas (REDISSEC), Zaragoza, Spain; 10 Fundación para el Fomento de la Investigación Sanitaria y Biomédica de la Comunitat Valenciana (FISABIO-Salud Pública), Valencia, Spain; 11 Health Services and Policy Research Group, Exeter Collaboration for Academic Primary Care, University of Exeter Medical School, University of Exeter, Exeter, UK; 12 Department of History of Science and Documentation, University of Valencia, Valencia, Spain; 13 Unidad de Información e Investigación Social y Sanitaria-UISYS, University of Valencia-Spanish National Research Council (CSIC), Valencia, Spain; 14 Instituto de Neurociencias de Alicante, Universidad Miguel Hernández-Spanish National Research Council (UMH-CSIC), San Juan de Alicante, Spain; 15 Directorate General for Public Health, Regional Health Council, Madrid, Spain; 16 Division of Pharmacoepidemiology and Pharmacovigilance, Spanish Medicines and Healthcare Products Agency (AEMPS), Madrid, Spain; 17 Department of Health Systems and Services, Unit of Medicines and Health Technologies, Pan American Health Organization (PAHO), Washington, DC USA; 18 Department of Psychiatry, Hospital Marqués de Valdecilla, University of Cantabria/IDIVAL and CIBERSAM, Santander, Spain; 19 Hospital Clínic, Universitat de Barcelona, Institut d’Investigacions Biomèdiques August Pi i Sunyer (IDIBAPS) and CIBERSAM, Barcelona, Spain; 20 Structural Biology and Biocumputing Programme, Spanish National Cancer Research Centre (CNIO), Madrid, Spain; 21 Life Sciences Department, Barcelona Supercomputing Center, Barcelona, Spain

**Keywords:** Cancer, Central nervous system disorder, Alzheimer’s disease, Anorexia nervosa, Amyotrophic lateral sclerosis, Autism spectrum disorders, Bipolar disorder, Depression, Down’s syndrome, Epilepsy, Huntington’s disease, Multiple sclerosis, Parkinson’s disease, Schizophrenia, Systematic review, Meta-analysis

## Abstract

**Background:**

The objective of this study will be to synthesize the epidemiological evidence and evaluate the validity of the associations between central nervous system disorders and the risk of developing or dying from cancer.

**Methods/design:**

We will perform an umbrella review of systematic reviews and conduct updated meta-analyses of observational studies (cohort and case-control) investigating the association between central nervous system disorders and the risk of developing or dying from any cancer or specific types of cancer. Searches involving PubMed/MEDLINE, EMBASE, SCOPUS and Web of Science will be used to identify systematic reviews and meta-analyses of observational studies. In addition, online databases will be checked for observational studies published outside the time frames of previous reviews. Eligible central nervous system disorders will be Alzheimer’s disease, anorexia nervosa, amyotrophic lateral sclerosis, autism spectrum disorders, bipolar disorder, depression, Down’s syndrome, epilepsy, Huntington’s disease, multiple sclerosis, Parkinson’s disease and schizophrenia. The primary outcomes will be cancer incidence and cancer mortality in association with a central nervous system disorder. Secondary outcome measures will be site-specific cancer incidence and mortality, respectively. Two reviewers will independently screen references identified by the literature search, as well as potentially relevant full-text articles. Data will be abstracted, and study quality/risk of bias will be appraised by two reviewers independently. Conflicts at all levels of screening and abstraction will be resolved through discussion. Random-effects meta-analyses of primary observational studies will be conducted where appropriate. Parameters for exploring statistical heterogeneity are pre-specified. The World Cancer Research Fund (WCRF)/American Institute for Cancer Research (AICR) criteria and the Grading of Recommendations Assessment, Development and Evaluation (GRADE) approach will be used for determining the quality of evidence for cancer outcomes.

**Discussion:**

Our study will establish the extent of the epidemiological evidence underlying the associations between central nervous system disorders and cancer and will provide a rigorous and updated synthesis of a range of important site-specific cancer outcomes.

**Systematic review registration:**

PROSPERO CRD42016052762

**Electronic supplementary material:**

The online version of this article (doi:10.1186/s13643-017-0466-y) contains supplementary material, which is available to authorized users.

## Background

Cancer caused over 8.8 million deaths worldwide in 2015 and has moved from the third leading cause of death in 1990 to the second leading cause in 2015 [1, 2]. Similarly, central nervous system disorders are a leading cause of the disease burden worldwide, substantially contributing to health loss across the lifespan [3–5]. Population growth and aging are producing a shift in the global burden of disease from communicable to chronic non-communicable diseases and from premature mortality to morbidity [4, 5]. This epidemiological transition is contributing to a rise in the global burden of chronic diseases such as central nervous system disorders and cancer, particularly in low-income regions [4–6]. In addition, comorbidity or multimorbidity (the presence of two or more chronic medical conditions in an individual) represents a paradigm shift from a single disease-based model to one that focuses on care for patients with multiple conditions [6–8].

During the past decades, a series of epidemiological observational studies and meta-analyses have claimed that central nervous disorders are associated with increased risk of cancer at several specific sites (e.g. an increased risk of brain cancer with multiple sclerosis, an increased risk of breast cancer with schizophrenia, an increased risk of melanoma with Parkinson’s disease, or an increased risk of leukaemia and testicular cancer with Down’s syndrome) [9–17]. At the same time, there is now epidemiological evidence suggesting a decreased risk of cancer in certain central nervous system disorders such as Alzheimer’s disease, multiple sclerosis and Parkinson’s disease [16–26]. In 2013, members of our review team conducted meta-analyses of cancer incidence in 50 observational studies including more than 570,000 participants from different settings (involving eight central nervous disorders such as Alzheimer’s disease, amyotrophic lateral sclerosis, autism spectrum disorders, Down’s syndrome, Huntington’s disease, multiple sclerosis, Parkinson’s disease and schizophrenia and eight site-specific cancers such as brain, breast, colorectal, lung, prostate, testicular cancers, leukaemia and melanoma) [16]. The main findings were published in 2014, suggesting that the presence of central nervous system disorders was associated with a decreased risk of cancer (risk ratio = 0.92; 95% confidence interval (CI) 0.87–0.98). Similarly, a decreased risk of cancer was detected in patients with neurodegenerative disorders (0.80; 95% CI 0.75–0.86) and in those with Alzheimer’s disease (0.32; 95% CI 0.22–0.46), Parkinson’s disease (0.83; 95% CI 0.76–0.91), multiple sclerosis (0.91; 95% CI 0.87–0.95) and Huntington’s disease (0.53; 95% CI 0.42–0.67). Patients with Down’s syndrome had an increased risk of cancer (1.46; 95% CI 1.08–1.96). We did not observe associations between cancer and amyotrophic lateral sclerosis or schizophrenia [16].

Many factors might account for these epidemiological associations, both biological, such as opposing genes and pathways, and non-biological, such as behaviors, diagnostic patterns or medication effects [17, 27, 28]. Because of its biological plausibility, the so-called ‘inverse cancer comorbidity’ in people with central nervous system disorders has captured the imagination and efforts of a growing number of scientists and researchers worldwide [16, 28]. In fact, cancer and central nervous system disorders share many genes and biological pathways, and these are often regulated in different directions [16, 17, 27–29]. However, it is also possible that some claimed epidemiological associations could be caused by chance, confounding and biases in the published biomedical literature. Potential biases have been discussed and suggested in the epidemiology of cancer for multiple risk factors, biomarkers and prognostic factors, including selective reporting bias (e.g. favoring the publication of significant associations) [30–34].

Past reviews [13–15, 22, 26] have considered some of the potential associations between cancer and specific central nervous system disorders in isolation. There is a need for comprehensive syntheses of all available epidemiological data in unified analyses evaluating the strength of the overall body of the evidence supporting causal connections for disease condition-outcome pairs. Data from epidemiological studies for some central nervous system disorders and cancer have been sparse in previous reviews [16, 28], and recently published data could also help to improve estimations [35–40]. Therefore, we consider it timely to update and expand on previous meta-analyses examining the inverse and direct cancer comorbidity in people with central nervous system disorders.

We will perform an umbrella review of systematic reviews and conduct updated meta-analyses of observational studies in order to synthesize all the available epidemiological evidence and evaluate the validity of the associations between central nervous system disorders and the risk of developing or dying from cancer.

## Methods

This protocol has been registered within the PROSPERO database for systematic reviews and meta-analyses (registration number: CRD42016052762). The protocol has been designed and reported in accordance with the reporting guidance provided in the Preferred Reporting Items for Systematic Reviews and Meta-Analyses Protocols (PRISMA-P) statement [41, 42] (see PRISMA-P checklist in Additional file 1).

### Design

We will conduct an umbrella review of systematic reviews and meta-analyses of observational studies in 12 central nervous system disorders (including Alzheimer’s disease, anorexia nervosa, amyotrophic lateral sclerosis, autism spectrum disorders, bipolar disorder, depression, Down’s syndrome, epilepsy, Huntington’s disease, multiple sclerosis, Parkinson’s disease and schizophrenia) that reported the risk of developing or dying from any cancer or specific types of cancer. An umbrella review is a systematic collection and assessment of multiple systematic reviews and meta-analyses done on a specific research topic [33, 34, 43–46].

### Participants/population

Our review targets all human participants (regardless of age or sex) available from observational studies irrespective of setting (inpatient, outpatient or mixed community setting). For participants with a central nervous disorder, we will use investigator-reported definitions according to accepted diagnostic criteria (e.g. ninth or tenth revisions of the International Classification of Diseases (ICD) or the third or fourth edition of the Diagnostic and Statistical Manual of Mental Disorders (DSM) criteria). Eligible central nervous system disorders will be Alzheimer’s disease (ICD-9: 331.0, 290.1; ICD-10: F00, G30), anorexia nervosa (ICD-9: 307.1, 307.54; ICD-10: F50.0-F50.1), amyotrophic lateral sclerosis (ICD-9: 335.20; ICD-10: G12.2), autism spectrum disorders (ICD-9: 299.0, 299.8; ICD-10: F84), bipolar disorder (ICD-9: 296-296.16, 296.4-296.99, 301.1-301.13; ICD-10: F06.3-F06.34, F30-F31.9, F34.0), major depression (ICD-9: 296.2-296.36, 311-311.9, V11.1, V11.2; ICD-10: F32-F33.9), Down’s syndrome (ICD-9: 758.0; ICD-10: Q90), epilepsy (ICD-9: 345-345.91; ICD-10: G40-G41.9, Z82.0), Huntington’s disease (ICD-9: 294.1, 333.4; ICD-10: F02.2, G10), multiple sclerosis (ICD-9: 340-340.9; ICD-10: G35-G35.9), Parkinson’s disease (ICD-9: 332-332.9; ICD-10: G20-G21.0, G21.2-G22.0) and schizophrenia (ICD-9: 295-295.95, 301.0, 301.2-301.22, V11.0; ICD-10: F06.2, F20-F23.9, F25-F29.9). Exclusion criteria will be animals, in vitro and in vivo experiments.

### Study outcomes

The primary outcomes will be cancer incidence and cancer mortality (all malignant neoplasms; ICD-9: 140-209; ICD-10: C00-C97) in association with a specific central nervous system disorder (as defined above—see the ‘Participants/population’ section). Secondary outcome measures will be site-specific cancer incidence and site-specific cancer mortality studied in association with a specific central nervous system disorder. Following the Global Burden of Disease Cancer Collaboration system classification [47], site-specific cancer will be defined in groups that include ICD codes pertaining to neoplasms (see Additional file 3).

### Types of study to be included

The types of study to be included are systematic reviews, meta-analyses and observational studies (case-control and cohort) in humans that examined the association between central nervous system disorders and risk of developing or dying from cancer. Randomized controlled trials are unavailable for our research question. We will exclude studies in which central nervous system disorders were not the exposure of interest and cancer incidence or mortality was not the outcome of interest. Systematic reviews, meta-analyses and observational studies that did not present study specific data (e.g. relative risks or odds ratios with 95% confidence intervals and number of cases of cancer/population) will be also excluded. We will not apply any year, language, or publication status restrictions in the selection of eligible studies.

### Search methods

We will systematically search PubMed/MEDLINE, EMBASE, SCOPUS and Web of Science to identify systematic reviews and meta-analyses of observational studies examining associations between cancer and central nervous system disorders.

The final search strategies will be defined by a senior information specialist and a clinical epidemiologist. Keywords related to central nervous system disorders, cancer and systematic reviews will be used. A draft search strategy for PubMed/MEDLINE database has been included in Additional file 2. A manual review of references from eligible systematic or narrative reviews will be also performed. In addition, the online databases will be checked for eligible observational studies that have been published outside the time frames of previous reviews. In particular, PubMed/MEDLINE will be searched to identify other additional observational studies using a compiled list of the unique PubMed/MEDLINE identification numbers of all relevant articles, and a related article search will be performed. This technique has been shown to be effective in identifying relevant studies, increases efficiency in study identification in the presence of an already large evidence base [48] and is being used as part of complex evidence syntheses [49–51]. We will also contact authors of primary publications and/or collaborators to check if they are aware of any studies we may have missed. For disease-outcome pairs where no systematic reviews are available, we will conduct a new systematic search of literature that will be prospectively reported in a new specific protocol, following standard methods described in this paper. Any amendments or modifications made in the protocols will be outlined and reported in the final papers.

### Screening and selection procedure

Two reviewers will screen all articles identified from the search independently. First, titles and abstracts of articles returned from initial searches will be screened based on the eligibility criteria outlined above. Second, full texts will be examined in detail and screened for applicability. Third, references of all considered articles will be hand-searched to identify any relevant report missed in the search strategy. Any disagreement between reviewers will be resolved by discussion to meet a consensus.

### Data collection process

From each eligible systematic review and meta-analysis, two reviewers will extract information independently on first author, year of publication, central nervous system disorder(s) and outcome(s) examined, number of included studies, reported summary meta-analytic estimates (including heterogeneity measures), additional analyses (e.g. subgroup analysis, meta-regression, sensitivity analysis) and bias assessment (e.g. quality or risk of bias of each study and publication bias). For each of the included observational studies, two reviewers will also extract the epidemiological design (cohort or case-control, prospective or retrospective), the country of study, the follow-up period, the setting (mixed, inpatient, outpatient or community), coverage (multi-center or single-center study), the general characteristics of participants (age, sex and ethnicity), the sample size, the outcomes of interest (including definitions and confounding factors that were taken into consideration), the number of cases and controls (in case-control studies) or the number of cases and population participants (in cohort studies) and/or the maximally adjusted relative risk (reported as odds ratio for case-control studies and hazard ratio or standardized incidence/mortality ratio for cohort studies) and 95% confidence intervals. We will use pre-designed forms that will be piloted initially on a small number of included reviews and observational studies. We will also contact authors of primary publications and/or collaborators for missing outcome data or unclear information.

### Quality and risk of bias assessment

The methodological quality and bias of reviews will be appraised using the Assessment of Multiple Systematic Reviews (AMSTAR) tool [52]. This tool consists of 11 items and has good face and content validity for measuring the methodological quality of systematic reviews. Each item within the instrument can receive 1 point, for a possible range of AMSTAR scores of 0 to 11. The AMSTAR instrument will be administered independently by two reviewers, and discrepant scores will be resolved by discussion and consensus.

The methodological quality and bias of primary epidemiological studies will be appraised using the Newcastle Ottawa Scale (NOS) for observational (e.g. cohort and case-control) studies [53]. Using the NOS tool, each study is judged on eight items, categorized into three groups: the selection of the study groups, the comparability of the groups and the ascertainment of either the exposure or outcome of interest for case-control or cohort studies, respectively. Stars are awarded for each quality item, and the highest quality studies are awarded up to nine stars. We will consider studies with 0–3, 4–6 and 7–9 stars to represent high, moderate and low risk of bias, respectively. The risk of bias for each observational study will be independently assessed by two reviewers. Discrepant scores will be resolved by discussion and consensus.

### Methods for evidence synthesis

The data from each systematic review’s characteristics and findings based on methodological quality will be used to build evidence tables. The data extracted and summarized will include specific details about the research question, search strategy, eligibility criteria, population (sample size and participant characteristics), studies (type and number), methods and outcomes of significance to the review question and results reported in previous reviews. We will not conduct a meta-analysis of systematic reviews in this umbrella review. Pooling the results of the included reviews is likely to introduce potential biases as it will give too much statistical power to primary observational studies included in more than one review, with the risk that a misleading estimate will be produced and that this will be overly precise [54]. Instead, data from primary observational studies (primary studies from previous reviews with new studies) will be used to perform updated meta-analyses. We will estimate the summary effect size and its 95% confidence interval using the inverse variance method based on the DerSimonian and Laird random-effects model [55]. The random-effects model is selected a priori to synthesize the epidemiological data, as it considers both within-study and between-study variation by incorporating the heterogeneity of effects into the overall analyses. If primary studies report results separately for men and women or other subgroups, we will combine the subgroup-specific estimates using a fixed-effects model to generate an estimate for both subgroups combined so that each study was represented only once in the analyses. We will evaluate heterogeneity by estimating the variance between studies using Cochran’s Q test [56] and *I* ^2^ statistic [57]. The Cochran Q test is obtained by the weighted sum of the squared differences of the observed effect in each study minus the fixed summary effect. The *I* ^*2*^ statistic is the ratio of variance between studies over the sum of the variances within and between studies and ranges between 0 and 100% (with values of 0–25%, 25–50%, 50–75% and 75–100% taken to indicate low, moderate, substantial and considerable heterogeneity, respectively). We will also estimate the 95% prediction interval [58, 59], which further accounts for between-study heterogeneity and evaluates the uncertainty for the effect that would be expected in a new observational study addressing that same association.

As a further important improvement over previous reviews and meta-analyses, we will apply a set of criteria to conclude whether the evidence for a given outcome may be considered convincing, probable, limited-suggestive, limited-not conclusive or unlikely. For this, we will follow the Global Burden of Disease Study approach [60, 61] using the World Cancer Research Fund (WCRF)/American Institute for Cancer Research (AICR) criteria for grading evidence [62]. ‘Convincing evidence’ consists of biologically plausible associations between exposure and outcome established from multiple epidemiological studies in different populations. Evidentiary studies must be substantial, must include prospective observational studies and, where relevant, randomized controlled trials of sufficient size, duration and quality and must show consistent effects. A convincing relationship should be robust enough to be highly unlikely to be modified in the foreseeable future as new evidence accumulates. ‘Probable evidence’ is similarly based on epidemiological studies with consistent associations between exposure and outcome, but shortcomings in the evidence exist, such as insufficient trials or prospective observational studies available. ‘Limited-suggestive evidence’ represents too limited evidence to permit a probable or convincing causal judgement, but where there is evidence suggestive of a direction of effect. ‘Limited-not conclusive evidence’ consists of information that is so limited that no firm conclusion can be made for a number of reasons (e.g. the evidence might be limited by the amount of evidence in terms of the number of studies available, by inconsistency of direction of effect, by poor quality of studies or by any combination of these factors). ‘Substantial effect on risk unlikely’ consists of evidence strong enough to support a judgement that a particular exposure is unlikely to have a substantial causal relation to a cancer outcome. The evidence should be robust enough to be unlikely to be modified in the foreseeable future as new evidence accumulates [60–62].

We will also use the Grading of Recommendations Assessment, Development and Evaluation (GRADE) methodology for evaluating the quality of evidence for each outcome [63]. GRADE rating will be adjudicated as high (further research is very unlikely to change our confidence in the estimate of effect), moderate (further research is likely to have an important impact on our confidence in the estimate of effect and may change the estimate), low (further research is very likely to have an important impact on our confidence in the estimate of effect and is likely to change the estimate) or very low (very uncertain about the estimate of effect).

### Additional analyses

Using random-effects meta-analyses, we will present combined effect sizes estimates for the cluster of neurodegenerative disorders (including Alzheimer’s disease, amyotrophic lateral sclerosis, Huntington’s disease, multiple sclerosis and Parkinson’s disease) taking into consideration the growing evidence suggesting that cancer and neurodegenerative diseases share genes and biological pathways—the *neurodegeneration hypothesis* [16, 27, 28].

If sufficient studies are identified, potential sources of heterogeneity will be investigated further by subgroup or meta-regression analyses according to baseline characteristics and methodological factors. We plan to conduct subgroup analyses by sex (men or women), study design (cohort or case-control; prospective or retrospective), follow-up (0–1, >1–5 or >5 years), setting (mixed, inpatient, outpatient or community), population based (yes or no), country economic status (developed or developing countries according to International Monetary Fund), year of publication (before 2000 or in 2000 and after), study quality (high or low-moderate risk of bias), adjustment for confounders (age, sex or other) and sample size (<500, 500–1000 or >1000 participants). If feasible, we will conduct not only a subgroup analysis by ethnicity (Asian or non-Asian) but also meta-regression analyses to investigate the potential impact of ethnic differences [39] by considering the percentage of Asian participants in epidemiological studies. We will conduct not only subgroup analyses for cancer types according to relationship with smoking (smoking-related cancer sites or other cancer sites) (see Additional file 3) but also meta-regression analyses considering the percentage of smokers (e.g. past or current smoker).

In order to further assess the consistency of evidence, we will perform cumulative meta-analyses in the order of publication year showing the pattern of evidence over time [64, 65]. Cumulative meta-analyses recognize the cumulative nature of scientific evidence and knowledge.

Publication bias or small study effects will be assessed by inspection of the funnel plots for asymmetry and with Egger’s test [66] and Begg’s test [67], with the results considered to indicate potential small study effects when *P* < 0.10.

### Software considerations

All analyses will be conducted in Stata version 13 or higher (StataCorp LP, College Station, TX, USA) using the metan (for fixed and random-effects meta-analysis), metareg (for meta-regression analysis), metacum (for cumulative meta-analysis) and metabias and metafunnel (for small study effects analysis) [68].

## Discussion

Evaluating and understanding the complex connections between cancer and central nervous system disorders might be of great importance for biomedical research, education, clinical practice and public health given the high burden of these disease conditions worldwide [1–5]. This review will establish the extent of the epidemiological evidence underlying the associations between central nervous system disorders and the risk of developing or dying from cancer. This protocol updates and will supersede previous meta-analyses of observational studies on this topic [16] and will provide a rigorous and updated synthesis of a range of important site-specific cancer outcomes. Any amendments made to this protocol when conducting the review will be outlined and reported in the final paper.

There are several strengths and limitations of our planned methods. We will comprehensively review and evaluate a significant amount of epidemiological data characterizing the associations between central nervous system disorders and cancer. Beyond summarizing the findings for a wide range of central nervous system disorders and cancers, we will explore the extent of bias and heterogeneity in the observational research. Furthermore, we anticipate that some outcomes will be poorly covered in the scientific literature, and thus, knowledge gaps will be identified. Our findings could potentially be useful not only for informing a research agenda of new epidemiological studies that can be linked to large world-class consortiums of scientists and researchers conducting prospective studies and meta-analyses but also for educational and training opportunities. A limitation is that based on knowledge from previous reviews, we anticipate identifying not only studies using different study designs, populations and outcome definitions (which may increase statistical heterogeneity in the potential associations) but also a lack of complete reporting of methods and results in some epidemiological studies [69–71]. Further, the possibility of selective outcome reporting bias in cancer epidemiology [30–34] could also be a potential limitation.

## Additional files

Additional file 1: PRISMA-P Checklist (DOCX 27 kb)

Additional file 2: Key terms for PubMed/MEDLINE search (DOCX 22 kb)

Additional file 3: Definitions of specific cancer-site outcomes (DOCX 26 kb)

## Abbreviations

AICR: American Institute for Cancer Research
AMSTAR: Assessment of Multiple Systematic Reviews
DSM: Diagnostic and Statistical Manual of Mental Disorders
GRADE: Grading of Recommendations Assessment, Development and Evaluation
ICD: International Classification of Diseases
NOS: Newcastle Ottawa Scale
PRISMA-P: Preferred Reporting Items for Systematic Reviews and Meta-Analyses extension for Protocols
WCRF: World Cancer Research Fund
