# Supplementary material for: Cancer and central nervous system disorders: protocol for an umbrella review of systematic reviews and updated meta-analyses of observational studies
Source: Syst Rev. 2017 Apr 4;6:69. doi: 10.1186/s13643-017-0466-y (PMC5379758; doi:10.1186/s13643-017-0466-y)
Supplement: Supplementary file 2 — Key terms for PubMed/MEDLINE search (DOCX 22 kb) [file 13643_2017_466_MOESM2_ESM.docx]

**Additional file 2**

**Key terms for PubMed/MEDLINE search.**

| **Search** | **Query** |
| --- | --- |
| #1 | **Search**alzheimer disease OR anorexia nervosa OR amyotrophic lateral sclerosis OR autism spectrum disorders OR autistic disorder OR bipolar disorder OR depressive disorder OR down syndrome OR epilepsy OR huntington disease OR multiple sclerosis OR parkinson disease OR schizophrenia |
| #2 | **Search** cancer OR carcinoma OR neoplasia OR tumor OR neoplasm OR maligna* |
| #3 | **Search** systematic review* OR systematic overview* OR evidence based review* OR evidence-based OR overview* OR meta-review* OR meta-analy* OR metanaly* OR metaanaly* OR metanaly* OR research overview* OR collaborative review* |
| #4 | **Search**  **#1 AND #2 AND #3**  **No limits** |
