# Supplementary material for: Cancer and central nervous system disorders: protocol for an umbrella review of systematic reviews and updated meta-analyses of observational studies
Source: Syst Rev. 2017 Apr 4;6:69. doi: 10.1186/s13643-017-0466-y (PMC5379758; doi:10.1186/s13643-017-0466-y)
Supplement: Supplementary file 3 — Definitions of specific cancer-site outcomes (DOCX 26 kb) [file 13643_2017_466_MOESM3_ESM.docx]

**Additional file 3**

**Table: Definitions of specific cancer-site outcomes.**

| **Specific cancer-site** | **ICD-9 code** | **ICD-10 code** |
| --- | --- | --- |
| Esophageal cancer^†^ | 150-150.9 | C15-C15.9 |
| Stomach cancer^†^ | 151-151.9, 209.23 | C16-C16.9 |
| Liver cancer^†^ | 155-155.3 | C22-C22.9 |
| Larynx cancer^†^ | 161-161.9, 162.1 | C32-C32.9 |
| Tracheal, bronchus and lung cancers^†^ | 162, 162.0, 162.2-162.9, 209.21 | C33-C34.9 |
| Breast cancer | 174-175.9 | C50-C50.929 |
| Cervical cancer^†^ | 180-180.9 | C53-C53.9 |
| Uterine cancer^†^ | 182-182.8 | C54-C54.9 |
| Prostate cancer | 185-185.9 | C61-C61.9 |
| Colon and rectum cancer | 153-154.9, 155.5, 155.9, 209.1-209.17 | C18-C20.0, C20.9-C21.8 |
| Lip and oral cavity cancer^†^ | 140-145.9 | C00-C08.9 |
| Nasopharynx cancer^†^ | 147-147.9 | C11-C11.9 |
| Other pharynx cancer^†^ | 146-146.9, 148-148.9 | C09-C10.9, C12-C13.9 |
| Gallbladder and biliary tract cancer | 156-156.9, 209.25-209.27 | C20.8, C23-C24.9 |
| Pancreatic cancer^†^ | 157-157.9 | C25-C25.9 |
| Malignant skin melanoma | 172-172.9 | C43-C43.9, C4A |
| Ovarian cancer^†^ | 183, 183.0 | C56-C56.9 |
| Testicular cancer | 186-186.9 | C62-C62.92 |
| Kidney cancer^†^ | 189.0, 189.1, 209.24 | C64-C65.9 |
| Bladder cancer^†^ | 188-188.9 | C67-C67.9 |
| Brain and nervous system cancer | 191-192.9 | C70-C72.9 |
| Thyroid cancer | 193-192.9 | C73-C73.9 |
| Mesothelioma | 58-158.9, 163-163.3, 163.8, 163.9 | C45-C45.9 |
| Hodgkin lymphoma | 201-201.98 | C81-C81.99 |
| Non-Hodgkin lymphoma | 200-200.9, 202-202.98 | C82-C86.6, C96-C97.9 |
| Multiple myeloma | 203-203.9 | C88-C90.32 |
| Leukaemia^†^ | 204-208.92 | C91-C95.92 |
| Other neoplams | 152-152.9, 160-160.9,164-164.9, 170-171.9, 181-181.9, 182.9, 183.2-183.8, 184.0-184.4, 184.8, 187.1-187.8, 189.2-189.8, 190-190.9, 194-194.8 | C17-C17.9, C30-C31.9, C37-C38.8, C40-C41.9, C47-C49.9, C50.12-C50.129, C51-C52.9, C57-C57.8, C58, C58.0, C60-C60.9, C63-C63.8, C66-C66.9, C68.0-C68.8, C69-C69.92, C74-C75.8 |
| Other benign neoplasm | 209.4, 209.40, 209.41, 209.42, 209.43, 211.2, 211.8, 212.0, 212.4, 212.5, 212.6, 212.7, 212.8, 213, 213.0, 213.1, 213.2, 213.3, 213.4, 213.5, 213.6, 213.7, 213.8, 213.9, 214.2, 214.3, 214.4, 214.8, 214.9, 221.0, 221.1, 221.2, 221.8, 222.1, 222.8, 223.2, 223.8, 223.81, 223.89, 224, 224.0, 224.1, 224.2, 224.3, 224.4, 224.5, 224.6, 224.7, 224.8, 224.9, 227, 227.0, 227.1, 227.3, 227.4, 227.5, 227.6, 227.8, 227.9, 228, 228.0, 228.00, 228.01, 228.02, 228.03, 228.04, 228.09, 228.1, 228.9, 229.0, 229.8, 230.7, 230.8, 233.31, 233.32, 233.4, 233.5, 234.0, 234.5, 234.8, 235.4, 235.8, 236.1, 236.99, 238.0, 238.1, 238.4, 238.5, 238.6, 238.7, 238.71, 238.72, 238.73, 238.74, 238.75, 238.76, 238.77, 238.79, 238.8, 239.2, 623.0, 623.1, 623.7, 210.8 | D07.1, D07.2, D07.4, D07.5, D09.2, D09.20, D09.21, D09.22, D10.7, D13.2, D13.3, D13.30, D13.39, D14.0, D15, D15.0, D15.1, D15.2, D15.7, D15.9, D16, D16.0, D16.00, D16.01,  D16.02, D16.1, D16.10, D16.11, D16.12, D16.2, D16.20, D16.21, D16.22, D16.3, D16.30, D16.31, D16.32, D16.4, D16.5, D16.6, D16.7, D16.8, D16.9, D17.9, D18, D18.0, D18.00, D18.01, D18.02, D18.03, D18.09, D18.1, D19, D19.0, D19.1, D19.7, D19.9, D20, D20.0, D20.1, D20.9, D21, D21.0, D21.1, D21.10, D21.11, D21.12, D21.2, D21.20, D21.21, D21.22, D21.3, D21.4, D21.5, D21.6, D21.9, D28.0, D28.1, D28.2, D28.7, D29.0, D30.2, D30.20, D30.21, D30.22, D30.4, D30.7, D30.8, D31, D31.0, D31.00, D31.01, D31.02, D31.1, D31.10, D31.11, D31.12, D31.2, D31.20, D31.21, D31.22, D31.3, D31.30, D31.31, D31.32, D31.4, D31.40, D31.41, D31.42, D31.5, D31.50, D31.51, D31.52, D31.6, D31.60, D31.61, D31.62, D31.9, D31.90, D31.91, D31.92, D35, D35.0, D35.00, D35.01, D35.02, D35.1, D35.2, D35.3, D35.4, D35.5, D35.6, D35.7, D35.8, D35.9, D36, D36.1, D36.10, D36.11, D36.12, D36.13, D36.14, D36.15, D36.16, D36.17, D36.7, D37.2, D38.2, D38.3, D38.4, D38.5, D39.2, D39.7, D39.8, D41.2, D41.20, D41.21, D41.22, D41.3, D44.1, D44.10, D44.11, D44.12, D44.2, D44.3, D44.4,D44.5,D44.6,D44.7,D44.8,D45,  D45.0, D45.9, D46, D46.0, D46.1, D46.2, D46.20, D46.21, D46.22, D46.3, D46.4, D46.5, D46.7, D46.9, D47, D47.3, D47.4, D47.5, D47.7, D48.0, D48.1, D48.2, D48.3, D48.4, D48.7, D49.81 |

Note: List of International Classification of Diseases (ICD) codes mapped to the Global Burden of Disease cause list for cancer.

^†^Smoking-related cancer sites: lip and oral cavity cancer, esophageal cancer, stomach cancer, liver cancer, pancreatic cancer, nasopharynx cancer, larynx cancer, lung cancer (tracheal, bronchus and lung cancers), ovarian cancer, cervical cancer, uterine cancer, kidney cancer, bladder cancer, leukaemia.
